# The value of low-intensity pulsed ultrasound in reducing ovarian injury caused by chemotherapy in mice

**DOI:** 10.1186/s12958-024-01216-8

**Published:** 2024-04-26

**Authors:** Yi Zhou, Fengyu Zhu, Yuanyuan Zhou, Xuqing Li, Shuhan Zhao, Yiqing Zhang, Ying Zhu, Hongyan Li, Yunxia Cao, Chaoxue Zhang

**Affiliations:** 1 Department of Ultrasound, The first Affiliated Hospital of Anhui Medical University, NO.218 Jixi Road, Shushan District, Hefei, Anhui Province 230022 China; 2 Department of Obstetrics and Gynecology, The first Affiliated Hospital of Anhui Medical University, NO.218 Jixi Road, Shushan District, Hefei, Anhui Province 230022 China

**Keywords:** Low-intensity pulsed ultrasound, Premature ovarian failure, Chemotherapy, Oxidative damage, Ovarian fibrosis, Inflammation

## Abstract

**Background:**

Ovarian damage and follicle loss are major side effects of chemotherapy in young female patients with cancer. However, effective strategies to prevent these injuries are still lacking. The purpose of this study was to verify low-intensity pulsed ultrasound (LIPUS) can reduce ovarian injury caused by chemotherapy and to explore its underlying mechanisms in mice model.

**Methods:**

The mice were randomly divided into the Control group, Cisplatin group, and Cisplatin + LIPUS group. The Cisplatin group and Cisplatin + LIPUS group were intraperitoneally injected with cisplatin every other day for a total of 10 injections, and the Control group was injected with saline. On the second day of each injection, the Cisplatin + LIPUS group received irradiation, whereas the other two groups received sham irradiation. We used a variety of biotechnologies to detect the differences in follicle count, granulosa cell apoptosis, fibrosis, transcriptome level, oxidative damage, and inflammation in differently treated mice.

**Result:**

LIPUS was able to reduce primordial follicle pool depletion induced by cisplatin and inhibit the apoptosis of granulosa cells. Transcriptomic results confirmed that LIPUS can reduce ovarian tissue injury. We demonstrated that LIPUS can relieve ovarian fibrosis by inhibiting TGF-β1/Smads pathway. Meanwhile, it can reduce the oxidative damage and reduced the mRNA levels of proinflammatory cytokines caused by chemotherapy.

**Conclusion:**

LIPUS can reduce the toxic effects of chemotherapy drugs on ovaries, inhibit ovarian fibrosis, reduce the inflammatory response, and redcue the oxidative damage, reduce follicle depletion and to maintain the number of follicle pools.

**Supplementary Information:**

The online version contains supplementary material available at 10.1186/s12958-024-01216-8.

## Introduction

With the influence of environmental, psychological and other factors, the incidence of cancer tends to be at younger ages [1, 2]. The number of new cases in female tumor patients aged 20–29 years and 30–39 years was significantly higher than that in male patients [3]. In addition, newer iterations of chemotherapy regimens and drugs have resulted in higher survival times for tumor patients while also causing side effects in other systems. As the first-line drug for a variety of solid tumors, cisplatin has a wide anticancer spectrum, strong action and synergistic effect with a variety of anticancer drugs, thus making it the most commonly used drug in combination chemotherapy. Meanwhile, cisplatin interferes with the transcription and replication of cells, activating apoptosis or autophagy of cells, and causing ischaemia, necrosis, inflammation and micro-vascular interstitial injury in female ovarian tissues [4, 5]. Chemotherapy can cause irreversible ovarian damage in the early stage, including premature ovarian failure (POF).

The manner of how to effectively protect fertility of female tumor patients has become an issue that must be resolved during the course of chemotherapy [6]. At present, hormone replacement therapy is clinically utilized, hormone supplementation can improve the symptoms of hot flashes, night sweats, joint pain and genital atrophy caused by low oestrogen. However, there is insufficient evidence to improve fertility, and hormone supplementation can cause other systematic side effects, as well as possibly inducing tumors [7]. In recent years, animal experiments have shown that the transplantation of mesenchymal stem cells (MSCs) can partially improve the structure and function of the damaged ovary and reduce ovarian interstitial fibrosis and granulosa cell apoptosis [8, 9]. However, the stem cells themselves are rejected by the host, and they are still in the preliminary stage of research concerning various shortcomings, such as the definition of safety and effective use, low viability after transplantation and limited differentiation potential [10–13]. The manner of how to maintain ovarian function and fertility of young female patients with POF after chemotherapy is an urgent problem to be solved.

Low-intensity pulse ultrasound (LIPUS) is defined as pulsed emission ultrasound that typically exhibits an acoustic intensity of less than 3 W/cm^2^, which produces sound pressure waves that can penetrate into living cells and cause a series of biochemical events at the cellular level [14]. This method can increase protein synthesis, promote the secretion of mastoid cells, change the migration function of fibroblasts, increase the intake of second messenger calcium ions and promote the secretion and release of various growth factors and anti-inflammatory molecules. As a result, this method has become a heavily researched topic. LIPUS ameliorates AngII-induced myocardial fibrosis by reducing inflammation through a caveolin-1-dependent pathway [15]. In terms of improving penile erectile dysfunction, LIPUS can improve male sexual function by downregulating the TGF-β1/Smad/CTGF signalling pathway in penile tissue [16]. Studies have shown that LIPUS can reduce ovarian toxicity caused by 4-vinylcyclohexene diepoxide, maintain follicle numbers and alleviate the occurrence of POF; however, the mechanisms and specific impacts of this process are unclear [17, 18]. Therefore, the purpose of this study was to verify whether LIPUS can improve ovarian injury in mice treated with chemotherapy and to explore its underlying mechanisms and specific impacts on this process.

## Methods and materials

All of the experimental procedures were approved by the Ethical Committee and the Institutional Animal Care and Use Committee (20,232,078).

### Reagents and antibodies

Cisplatin (HY-17,394) was purchased from MCE (New Jersey, USA). The TUNEL ApoGreen Detection Kit (40307ES20), the 1st Strand cDNA Synthesis Kit (11141ES60) and Hieff qPCR SYBR Green Master Mix (11201ES08) were purchased from YEASEN Biotechnology (Shanghai, China). The following primary antibodies were used in this study: anti-Bax (380,709), anti-collagen I (501,352), anti-α-SMA (380,653), anti-NOX4 (380,874), anti-TGF-β1(346,599), anti-p-smad2 (R26361), anti-p-smad3 (380,775), anti-smad2 (200,790), anti-smad3 (R25743). anti-beta tubulin (380,628), and anti-GAPDH (380,626), which were purchased from Zen BioScience (Chengdu, China). Rabbit anti-PCNA polyclonal antibody (bs-2007R) was obtained from Bioss (Beijing, China). Masson (G1340) and Sirus red stain (G1472) were obtained from Solarbio (Beijing, China). 8-OHdG (bs-1278R), 4HNE (bs-6313R), and 3NIT (bs-8551R) were purchased from Bioss (Beijing, China). Endogenous peroxidase blocker was obtained from Zhongshan Jinqiao (Beijing, China). Orthofluorescence microscope was purchased from ZEISS (Germany).

### Animals

A total of 42 SPF female ICR mice (weight of 30 ± 2 g and age of 7–8 weeks) were used in this study and were obtained from the Experimental Animal Center of Anhui Medical University. All of the animals were housed in a relatively stable environment with a cycle of lights on at 8 a.m. and off at 8 p.m., as well as maintained room temperature (21–25 ℃) with water and food available ad libitum. For adaptation to the new environment, the mice were handled for a one-week acclimatization period before the experiment.

### POF model establishment

Cisplatin is often used as an inducer for modelling animals with chemotherapy-induced premature ovarian failure, and we also adopted cisplatin to establish the model. Mice were randomly divided into three groups: Cisplatin + LIPUS group (*n* = 14), Cisplatin group (*n* = 14) and Control group (*n* = 14). To better simulate the chemotherapy process, the mice in the Cisplatin group and Cisplatin + LIPUS group were intraperitoneally injected with cisplatin (2 mg.kg^− 1^) every other day for a total of 10 injections, and the mice in the Control group received an equal volume of saline.

### Protocol of LIPUS treatment

**Fig. 1:**
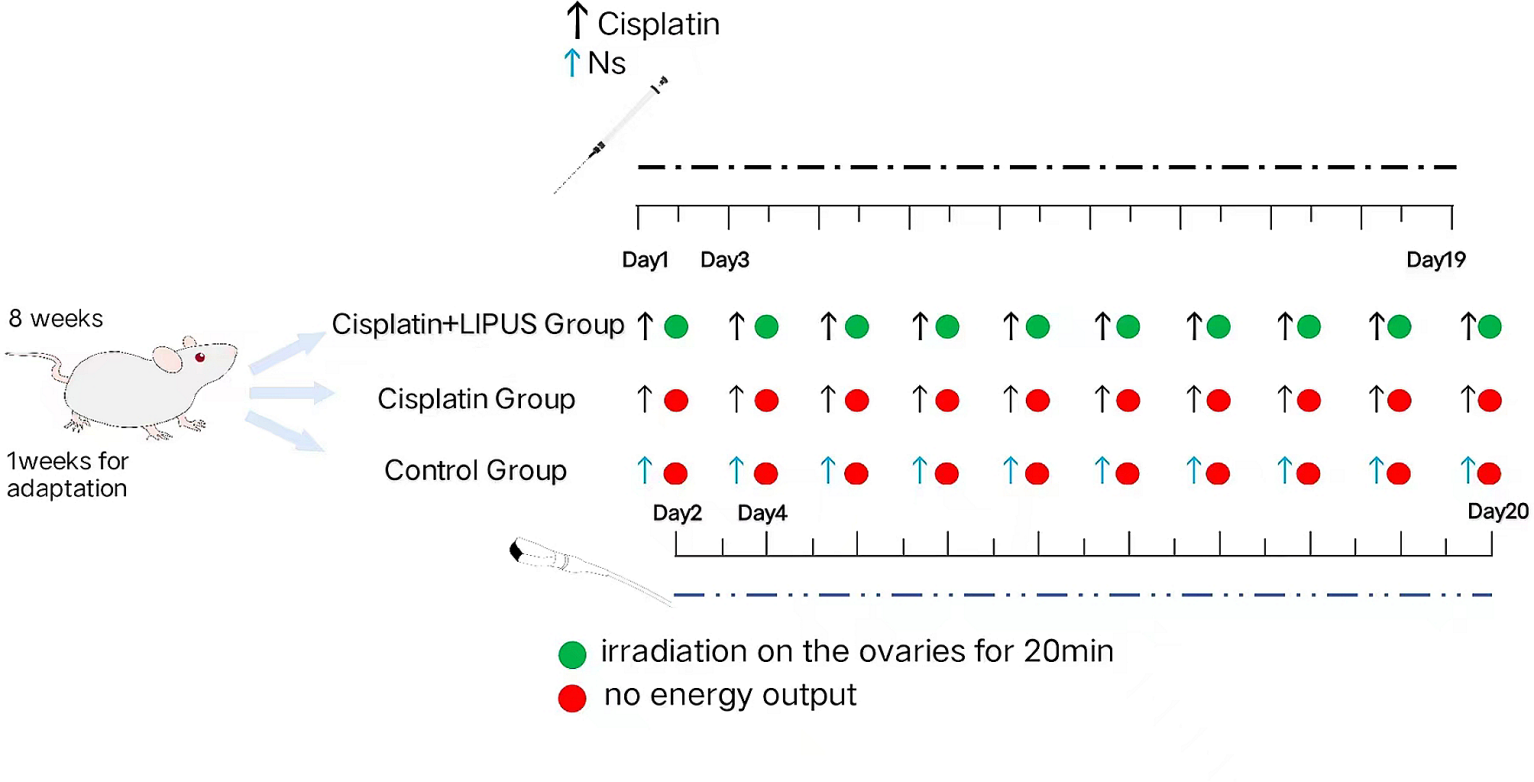
Flow chart of the experiment

The LIPUS device (Chongqing Haifu Medical Technology Co. LTD, Chongqing, China, RH2022D08) was used in this study. The following parameters were used in this study: the area of the transducer was 5 mm², the frequency of the transducer was 0.5 MHz, the duty cycle was 20%, and the spatial average temporal average intensity (*I*SATA) was 30 mW/cm^2^ [17]. Before the LIPUS treatment, the skin of the lower back and the bilateral ovaries were depilated, disinfected, and fully covered with the ultrasonic coupling agent. To better simulate ovarian protection, we performed ultrasound treatment throughout the course of chemotherapy. For the mice in the Cisplatin + LIPUS group, ultrasound treatment was performed on the second day after each injection, and each ovary was irradiated for 20 min until the next day after the last injection. The other two groups were handled in the same manner as the Cisplatin + LIPUS group except for a lack of energy output from LIPUS, the day after the last LIPUS treatment, the mice were sacrificed via cervical dislocation. (Fig. 1).

### Haematoxylin and eosin (H&E) staining and follicle count

The day after the last LIPUS treatment, the mice were sacrificed via cervical dislocation, and their bilateral ovaries were completely removed. The ovarian tissue was fixed and embedded in paraffin solution. A microtome was used to make consecutive sections with a thickness of 5 μm, and the slides were dewaxed. Subsequently, the structure of the ovarian tissue was identified by using haematoxylin and eosin staining. Subsequently, the slides were observed under an optical microscope. Follicle stages were classified according to Pederson’s standard. Specifically, an oocyte surrounded by a single layer of flattened or cubical granulosa cells was defined as a primordial or primary follicle; an oocyte surrounded by more than one layer of cuboidal granulosa cells without a visible antrum was defined as a secondary follicle; and a follicle possessing a clear antral space containing follicular fluid was defined as an antral follicle. For the follicle number, we counted every other fifth slice (e.g., 1, 6, 11). To avoid double counting, only nucleated follicles were counted.

### TdT-mediated dUTP Nick-End labeling (TUNEL) assay

The ovarian tissue sections were dewaxed, washed once with PBS, and then treated with 20 µg/ml protease K for cell penetration at room temperature for 10 min. The protease K was washed with PBS, and the TUNEL test solution prepared with 50 µl was added, incubated for 60 min at 37 °C away from light, and washed with PBS 3 times, after which the slices were sealed with anti-fluorescence quenching sealing solution and observed under a fluorescence microscope. At least 3 biological experiments were repeated.

### Masson and sirus red staining

Masson and Sirus red staining were used to detect fibrosis, and three slices from different ovaries were randomly selected to repeat the experiment. The percentage of positive signal area was analysed via Image J software, and statistical bar charts were drawn. Masson staining: the sections were dewaxed, successively stained with Weigert haematoxylin core, lichen red acid red solution, phosphomolybdic acid differentiation, and aniline blue drop staining, after which the slices were dipped, washed, dried and sealed. Sirus red stain: After dewaxing, the slices were stained successively with weigert hemithylin, differentiated with acidic differentiation liquid, stained with azure scarlet stain, washed and dried and sealed.

### Quantitative real-time reverse transcriptase PCR (RT‒qPCR)

Inflammatory cytokine (*IL-1β, NF-κB, Ccl2,IL-10* and *Tnf)* mRNA expression levels were assessed by using quantitative real-time reverse transcriptase PCR (RT‒qPCR). After the mice were sacrificed via cervical dislocation, the ovarian tissues were removed, and the TRleasyTM total RNA extraction kit was used in combination with isopropyl alcohol, 75% alcohol and gasform to extract RNA samples. A NANODROP 2000 was used for the quantitative analysis of RNA purity, and Hifairll 1stStrand cDNA Synthesis SuperMix for qPCR (gDNAdigester plus) Kit was used according to the manufacturer’s instructions. Subsequently, the RNA was reverse-transcribed into cDNA, and the obtained cDNA was diluted to an appropriate concentration by using the Hieff qPCR SYBR Green MasterMix (No Rox) kit according to the manufacturer’s instructions. For RT-qPCR, a real-time quantitative Lightcycler480 ll was utilized for light signal detection.

### Immunohistochemistry (IHC)

IHC for the detection of Bax, Bcl-2, α-SMA, Collagen I, TGF-β1, p-smad2, p-smad3, 4HNE, 8OHdG, and 3NIT was performed by using the peroxidase-labelled streptavidin-biotin method. Specifically, the slides were dewaxed and boiled in citrate buffer solution for 10 min for antigen retrieval. After cooling at room temperature, the sections were incubated with primary polyclonal rabbit antibodies against Bax, Bcl-2, α-SMA, Collagen I, TGF-β1, p-smad2, p-smad3, 4HNE, 8OHdG, and 3NIT. Each antibody was used at a dilution of 1:200, and antibodies were incubated for 2 h at room temperature. Immunostaining was completed by using diaminobenzidine (DAB) as a chromogen, and nuclear counterstaining was performed with Mayer’s haematoxylin. All of the slides were visualized by using a light microscope. At least three slices of unilateral ovarian tissue from different mice in each group were randomly selected to repeat the experiment.

### Western blot analysis (WB)

After the rats were euthanized in each group, the ovary tissues were rapidly excised and washed in a cold sterile 0.9% NaCl solution. Proteins were extracted and separated via 12% sodium dodecyl sulfate‒polyacrylamide gel electrophoresis, and the product was transferred to a polyvinylidene fluoride membrane, which was blocked with nonfat powdered milk for 1 h and subsequently incubated overnight at 4 °C with primary antibodies. After 4 washes for 10 min each with Tris-buffered saline Tween, secondary antibodies were added to the membrane and incubated for 1 h at room temperature. WB bands were evaluated by using the imaging system, and the grey values of the target bands were analysed by using ImageJ software.

### Immunofluorescence

After the slices were dewaxed, high-temperature antigen repair was performed. After natural cooling, endogenous peroxidase blocker was added and incubated for 10 min. Subsequently, the blocker was washed with PBS, and the primary antibody was added and incubated at 4 °C overnight. The next day, the primary antibody was discarded. After washing with PBS, the fluorescent rabbit secondary antibody was added and incubated for 1 h in the dark. After washing, the secondary antibodies were removed, the nuclei were stained with Hoechst, and the stained tissues were photographed under an orthofluorescence microscope. ImageJ software was used for image processing and analysis.

### Transcriptome sequencing

Total RNA quality detection, mRNA enrichment, mRNA interruption, double-stranded cDNA synthesis, terminal repair, additions of A and splice, fragment selection, PCR amplification, library quality assessment, and sequencing by Illumina were performed in different biological samples of each group. Quantitative analysis of the gene expression level was performed for each sample, and statistical analysis was conducted after the quantitative analysis was completed to screen the significantly differentially expressed genes in the samples under different states. Mapping of volcano plots and Venn diagrams based on upregulated and downregulated genes were also performed. The differential genes of all of the groups were collected and combined as differential gene sets. Moreover, the FPKM values of genes were analysed via hierarchical clustering, and a heatmap was drawn. GO analysis software GO-seq was used for the enrichment analysis to draw the bar graphs.

### Statistical analysis

At least three biological replicates were used to compute the means and SD. To compare significant differences between the groups, one-way analysis of variance using GraphPad Prism 8 (GraphPad Software Inc., San Diego, CA) was performed. To control for type I errors, all results were corrected using the Bonferroni method. *P* < 0.05 was considered to be statistically significant.

## Results

### LIPUS maintains the primordial follicle pool in cisplatin-treated mice ovaries

**Fig. 2:**
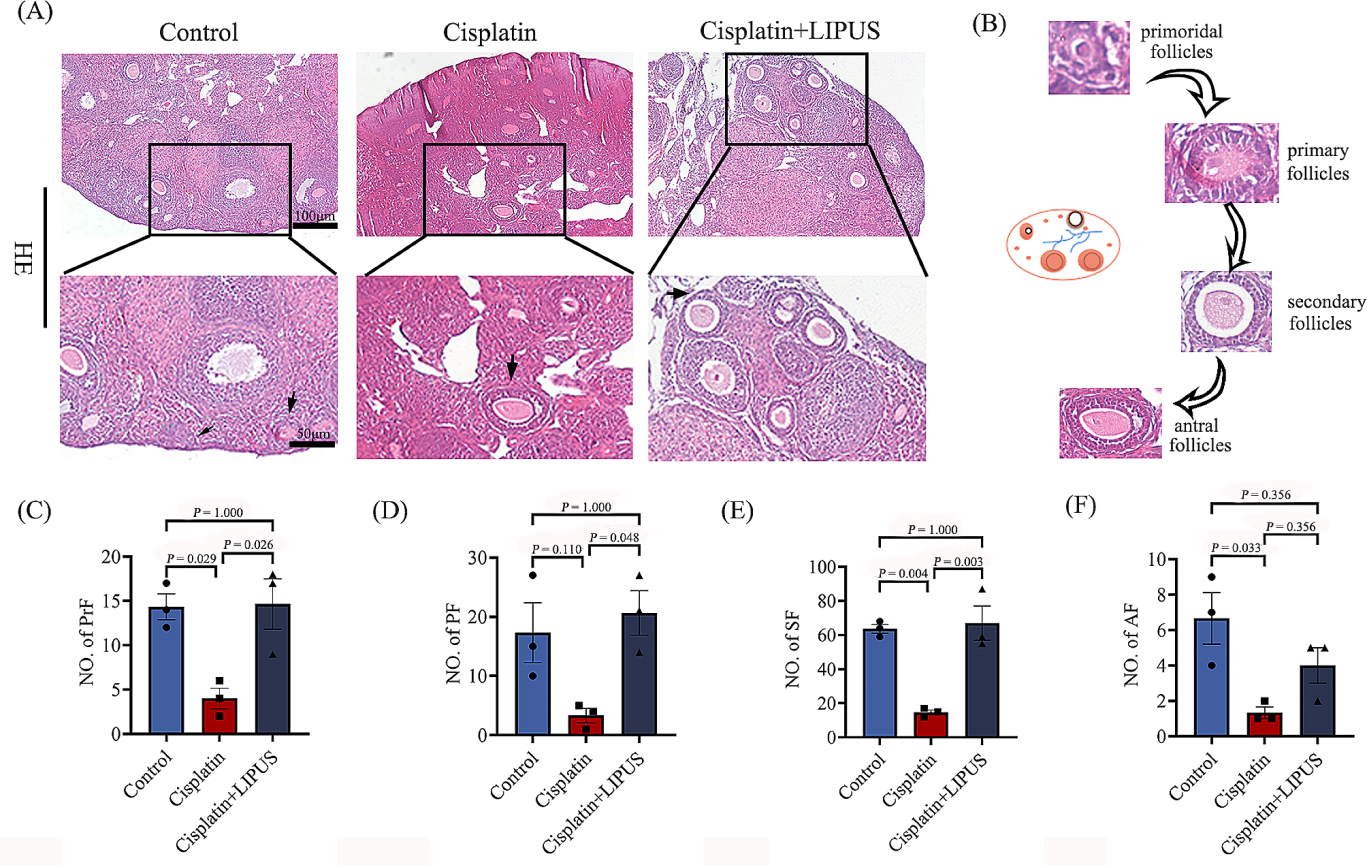
HE staining, follicle counting of ovarian tissue in three groups (100 μm and 50 μm), and statistical bar chart. Primordial follicles(PrF), primary follicles(PF), secondary follicles(SF), antral follicles(AF). (All results were corrected by Bonferroni method, and *P* < 0.05 after correction was considered to be statistically significant); *n* ≥ 3

To evaluate the success of our modelling methods and the effect of LIPUS on follicular development, we observed ovarian morphology via HE staining and performed follicle counting. The results showed that the ovaries in the Cisplatin group were irregular and significantly atrophied compared with those ovaries in the other two groups (Fig. 2A). Ovarian tissue atrophy was not obvious in mice treated with LIPUS, and the total number of follicles and the number of follicles at each stage were significantly higher than those of cisplatin mice, which was similar to that of the Control group (Fig. 2C-E); however, there was no significant difference in the number of antral follicles between the Cisplatin group and Cisplatin + LIPUS group (Fig. 2F). The results suggested the success of our model, and it has also been demonstrated that LIPUS treatment can reduce the depletion of follicle number caused by chemotherapy drugs and maintain the number of follicle pools.

### LIPUS suppresses the apoptosis of granulosa cells

**Fig. 3:**
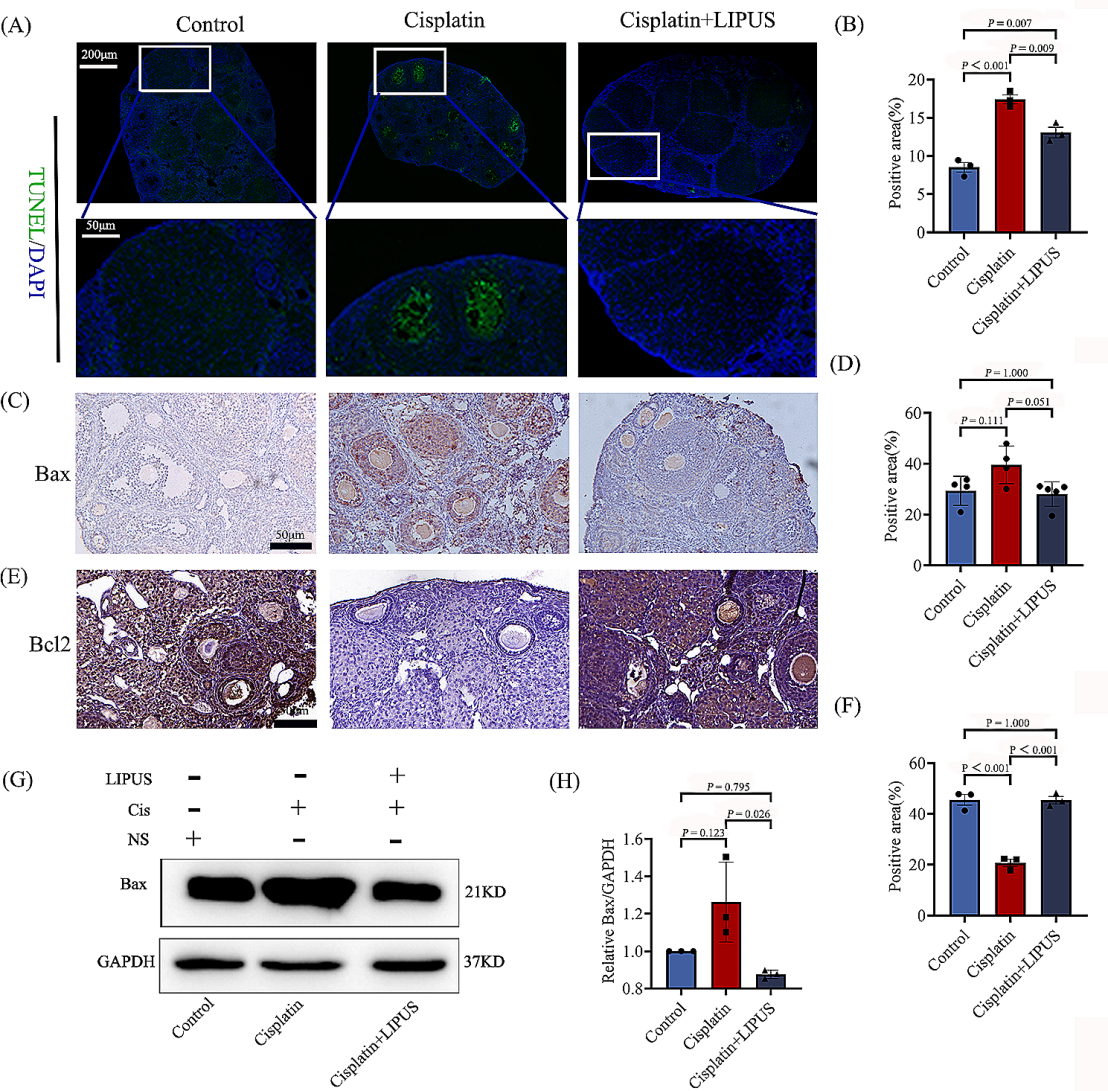
The apoptosis of ovarian tissue in three groups. (**A**): TUNEL fluorescence staining of the three groups. Green indicates positive staining areas (100 μm and 50 μm). (**B**): Statistical histogram of TUNEL staining. (**C**): The expression of Bax in ovarian tissues of the three groups was detected via IHC. Brown indicates positive staining areas (50 μm). (**D**): Statistical histogram of Bax by IHC. (**E**): The expression of Bcl-2 in ovarian tissues of the three groups was detected via IHC. Brown indicates positive staining areas (50 μm). (**F**): Statistical histogram of Bcl-2 by IHC. (**G**-**H**): The expression of Bax in ovarian tissues of the three groups was detected by using Western blotting, and a statistical histogram was conducted (All results were corrected by Bonferroni method, and *P* < 0.05 after correction was considered to be statistically significant); *n* ≥ 3

To verify that LIPUS can inhibit the apoptosis of granulosa cells, we performed a TUNEL assay of ovarian tissues from three groups of mice that were differentially treated. The results showed that the apoptosis rates of granulosa cells and follicles in the cisplatin mice were significantly higher than that in the Control group, and the apoptosis rate decreased significantly after LIPUS treatment (Fig. 3A-B). Moreover, Bax and Bcl-2 expression were detected by using immunohistochemistry. After cisplatin injection, the expression of Bcl-2 was significantly decreased, and the increase after LIPUS treatment was similar to that of the Control group (Fig. 3E-F). The result of Bax was opposite to that of Bcl-2,but the difference is not significant (Fig. 3C-D). To quantitatively analyse the expression of Bax at the protein level, WB detection was also performed, and the results showed that Bax expression in the ovarian tissues of the Cisplatin group was considerably increased compared to the Cisplatin + LIPUS group (Fig. 3G-H). We concluded that LIPUS could inhibit the apoptosis of granulosa cells and improve ovarian reserve in Cisplatin-induced mice.

### Transcriptomic results confirmed that LIPUS can improve ovarian tissue injury caused by chemotherapy

**Fig. 4:**
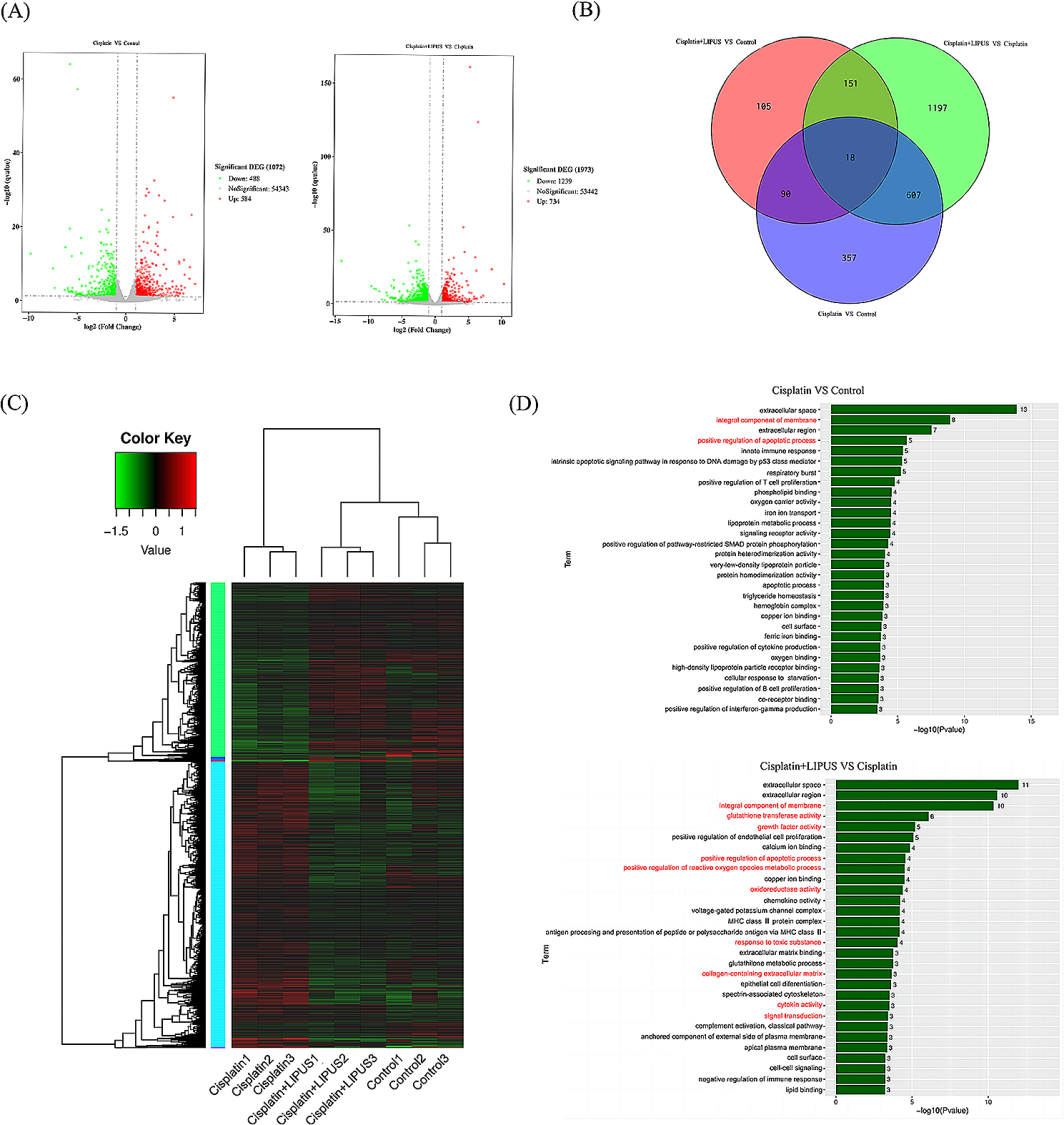
Transcriptome sequencing and bioinformatics analysis of three groups of mice with different treatments. (**A**): Volcanic maps of differentially expressed genes in the cisplatin and control groups and the Cisplatin and Cisplatin + LIPUS groups. Green indicates downregulated genes, and red indicates upregulated genes. (**B**): Venn diagram of different groups. (**C**): Heatmap. Different coloured areas represent different clustering group information. The Log10 (FPKM + 1) value was used for clustering, with red indicating high expression genes and green indicating low expression genes. The colours ranged from green to red, thus indicating higher gene expression. (**D**): GO analysis bar chart. GO enrichment *P* value histogram of differentially expressed genes in the Control vs Cisplatin groups and the Cisplatin vs Cisplatin + LIPUS groups. The ordinate is the enrichment GO term, and the abscise is the term-log10 (*P* value) value; *n* ≥ 3

To better understand how LIPUS reduce ovarian injury in chemotherapy mice and the underlying mechanisms, we conducted transcriptome sequencing and bioinformatics analysis. According to the expression of different genes in different groups, we generated a volcano map (Fig. 4A). Compared with the Control group, 584 up-regulated genes and 488 down-regulated genes were observed in the Cisplatin group; after LIPUS treatment, 734 up-regulated genes and 1,239 down-regulated genes were significantly different from those in the Cisplatin group. Moreover, we drew a Venn diagram to show the number of differentially expressed genes and co-expressed genes expressed between the different groups of ovaries (Fig. 4B). We performed a cluster analysis of differentially samples, a heatmap was drawn (Fig. 4C). Different coloured areas represent different clustering group information, and the analysis showed that the Cisplatin + LIPUS group and the Control group had more similar gene expression levels. In addition, we performed GO analysis for significantly differentially expressed genes (Fig. 4D). The differentially expressed genes were mainly concentrated in the following functional regions: integral component of membrane, glutathione transferase activity, growth factor activity, positive regulation of apoptotic process, positive regulation of reactive oxygen species metabolic process, oxidoreductase activity, response to toxic substance, collagen-containing extracellular matrix, cytokine activity, and signal transduction. Via transcriptome sequencing and bioinformatics analysis, it was confirmed that LIPUS could reduce ovarian damage caused by chemotherapy drugs and protect ovarian function.

### LIPUS inhibited ovarian fibrosis in mice treated with chemotherapy

**Fig. 5:**
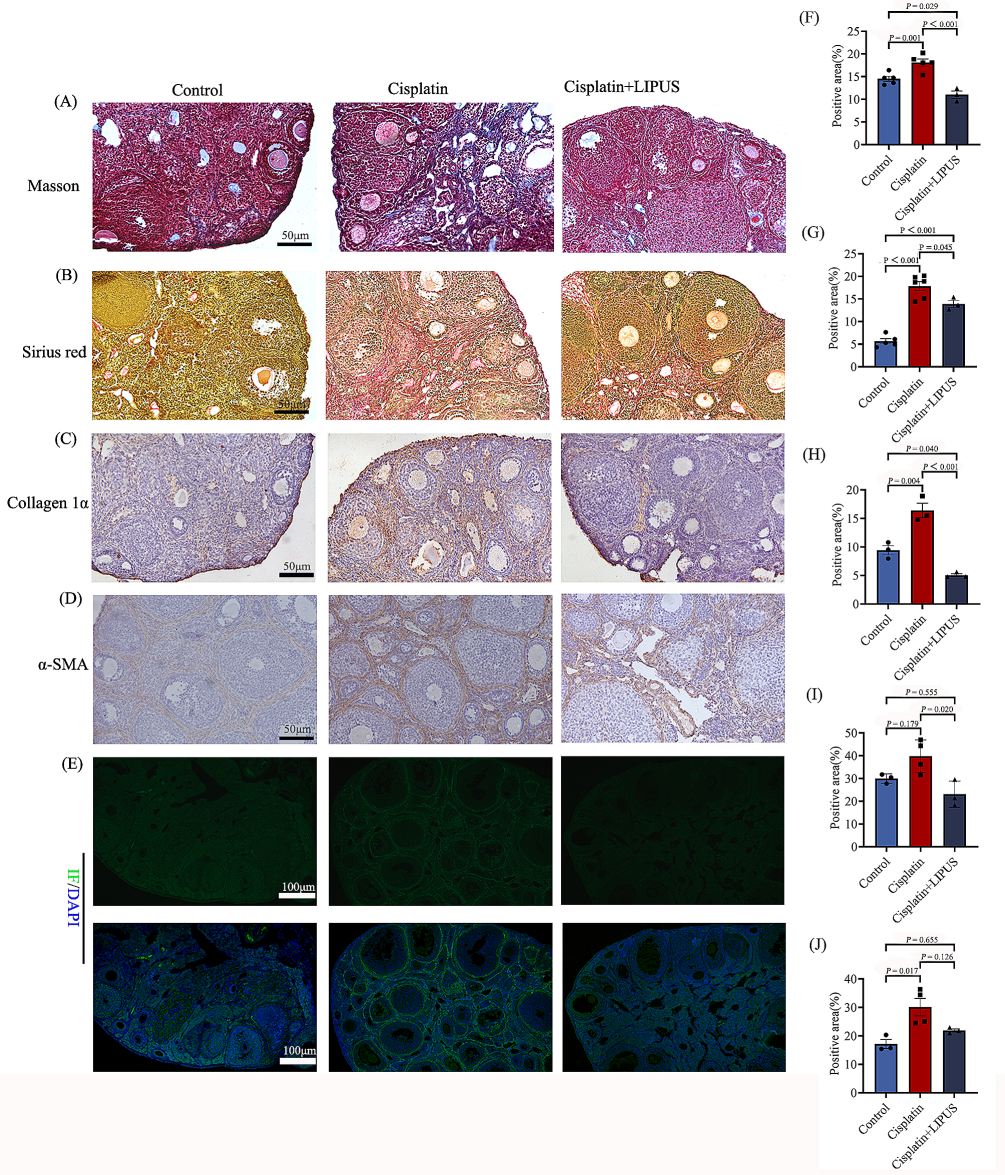
Fibrosis-related detection in three groups of mice. (**A**): Three groups of Masson staining. Blue areas indicate fibrosis (50 μm). (**B**): Three groups of Sirius red staining, the red area indicate fibrosis. (**C**-**D**): IHC detection of α-SMA and collagen I in the three groups (50 μm). (**E**): Immunofluorescence detection of α-SMA in ovarian tissue of mice in three groups. Green indicates positive areas (100 μm). (**F**): Statistical histogram of Masson staining. **G**: Statistical histogram of sirius red staining. H: Statistical histogram of positive staining for Collagen I. (**I**): Statistical histogram of positive staining for α-SMA. J: Statistical analysis of α-SMA immunofluorescence staining. (All results were corrected by Bonferroni method, and *P* < 0.05 after correction was considered to be statistically significant); *n* ≥ 3

It has been reported that cisplatin treatment causes ovarian fibrosis [7]. At the same time, we also found differences in fibrosis-related gene expression in transcriptome sequencing. To demonstrate that LIPUS can improve ovarian fibrosis in mice undergoing chemotherapy, we conducted Masson and Sirus red staining, and the results showed that the proportion of staining intensity and positive areas in the Cisplatin group was significantly higher than that in the Control group and Cisplatin + LIPUS group, that is, the cisplatin group had more collagen fiber deposition in the ovarian stroma and around the follicles (Fig. 5A-B and F-G). Moreover, ovarian fibrils levels were further confirmed by using IHC staining with α-smooth muscle actin (α-SMA) and Collagen I, which are the classic markers of fibrosis. As shown in Fig. 5C-D, the positive region in the Cisplatin group was greater than that in the Control group, whereas the Cisplatin + LIPUS group had a significantly reduced positive region compared with the Cisplatin group (Fig. 5H-I). The results of α-SMA immunofluorescence showed that there was a decreasing trend of ovarian fibrosis after low intensity ultrasound treatment, but there was no statistical significance (Fig. 5E and J).

### Identified of the related antibodies on TGF-β1/Smad pathway by IHC and WB

**Fig. 6:**
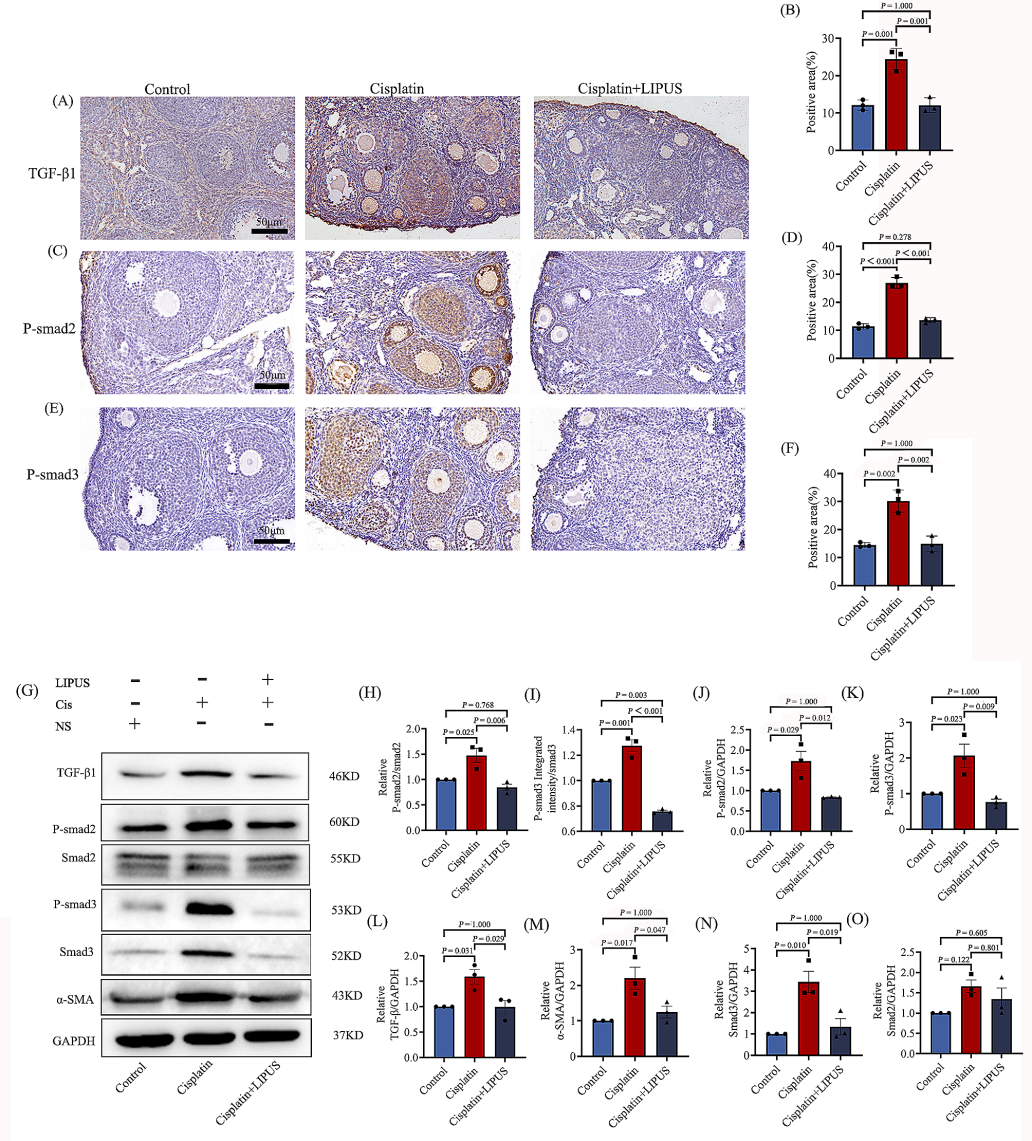
IHC and WB were used to verify the mechanisms associated with fibrosis. (**A**-**B**): IHC detection of TGF-β1in three groups (50 μm) and statistical bar chart. The brown area indicate positivity area. (**C**-**D**): IHC detection of p-smad2 in three groups (50 μm) and statistical bar chart. The brown area indicate positivity area. (**E**-**F**): IHC detection p-smad3 in three groups (50 μm) and statistical bar chart. The brown area indicate positivity area. (**G**): The expressions of TGF-β1, p-smad2, p-smad3, smad2, smad3, a-SMA and GAPHD in the ovaries of each group are analyzed by Western blot. (**H**-**O**): Quantitative analysis of protein expression of p-smad2/smad2, p-smad3/smad3,p-smad2, p-smad3, TGF-β1, a-SMA, smad2, and smad3. (All results were corrected by Bonferroni method, and *P* < 0.05 after correction was considered to be statistically significant); *n* ≥ 3. LIPUS = low-intensity pulsed ultrasound

Given that TGF-β1/Smad pathway is strongly associated with ovarian fibrosis [19].In order to investigate the mechanism of LIPUS alleviated ovarian fibrosis, we detected the TGF-β1, p-smad2, p-smad3 level in the ovary by IHC staining. In terms of positive staining area and intensity, cisplatin injection was significantly stronger than the Control group. In the Cisplatin group, positive staining was concentrated around the follicle, in the ovarian stroma. The Cisplatin + LIPUS group was significantly reduced compared with the Cisplatin group (Fig. 6A-F). IHC results confirmed LIPUS delayed ovarian fibrosis in cisplatin mice by inhibiting TGF-β1/Smad pathway.

To quantitatively analyze TGF-β1/Smad pathway, we detected TGF-β1, p-smad2, p-smad3, smad2, smad3 in protein level by WB. And results demonstrated that TGF-β1 and phosphorylated form of smad2 and smad3 was elevated after cisplatin treated alone, whereas this increase was significantly reduced by co-treatment with LIPUS, which was consistent with the results of IHC. The relative level of smad2 was not significantly different between control and treatment groups (Fig. 6G-L and N-O). In addition, we analyzed the α-SMA level in the Control group, Cisplatin group and Cisplatin + LIPUS group. Consistent with IHC staining, the α-SMA level in Cisplatin group was higher than Cisplatin + LIPUS (Fig. 6G and M). These results further suggest that cisplatin-induced ovarian fibrosis may be mainly associated with phosphorylation and activation of TGF-β1/Smad pathway components. Furthermore, LIPUS has a protective effect on cisplatin-induced ovarian fibrosis by blocking the activation of TGF-β1/Smad pathway components.

### LIPUS can reduce oxidative damage to mouse ovaries caused by chemotherapy drugs

**Fig. 7:**
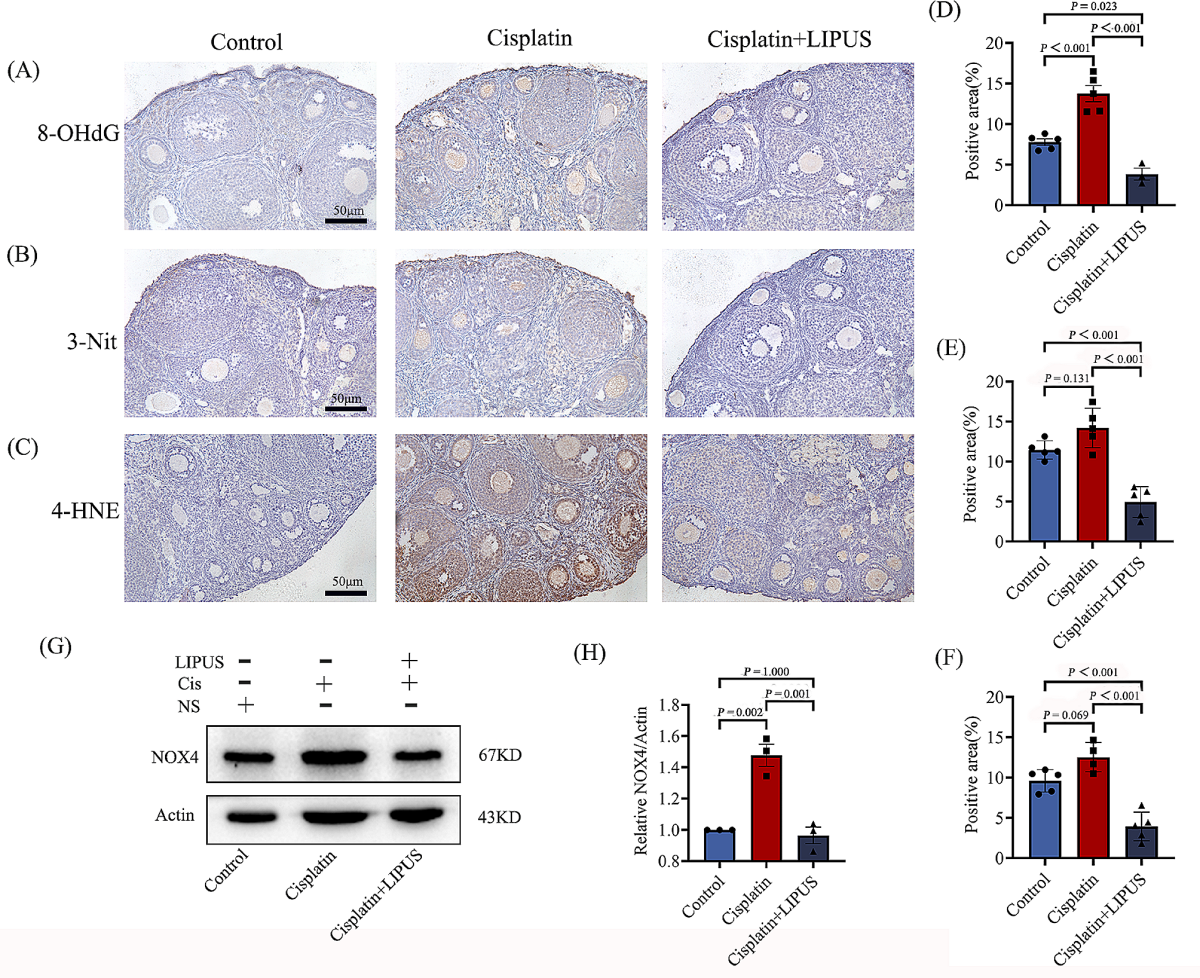
Correlation detection of ovarian tissue oxidative damage in three groups of mice. (**A**-**C**): IHC detection of 8-OHdG, 3-NIT, and 4-HNE (50 μm). The brown area indicates the positivity area. (**D**): Statistical histogram of positive staining for 8-OHdG (**E**): Statistical histogram of positive staining for 3-NIT. (**F**): Statistical histogram of positive staining for 4-HNE. (**G**-**H**): WB detection and statistical analysis of NOX4 in ovarian tissue of three groups of mice (All results were corrected by Bonferroni method, and *P* < 0.05 after correction was considered to be statistically significant); *n* ≥ 3

In the transcriptome sequencing analysis, we found significant differences in genes related to oxidative stress. Meanwhile, it has also been reported that oxidative damage caused by chemotherapy drugs can lead to fibrosis [20]. To confirm that LIPUS can reduce oxidative stress damage in mice undergoing chemotherapy, we detected the level of 8OHdG, 3NIT and 4HNE via IHC. The results showed that after cisplatin injection, the ovarian tissue of mice was damaged by oxidation, and the positive staining area and intensity were higher than those of the Control group. After LIPUS treatment, oxidative damage was alleviated (Fig. 7A-F). We also conducted quantitative detection of protein expression and detected the expression of NOX4. NOX4, which is a major contributor to oxidative stress, is a major source of ROS [21]. The results showed that the NOX4 expression level in mice treated with chemotherapy alone was higher than that in the other two groups, and NOX4 expression was significantly reduced in the ovarian tissue of mice treated with both chemotherapy and LIPUS. The results confirmed that LIPUS can alleviate the oxidative damage to mouse ovaries caused by chemotherapy drugs (Fig. 7G-H).

### LIPUS reduced ovarian inflammation in mice treated with chemotherapy

**Fig. 8:**
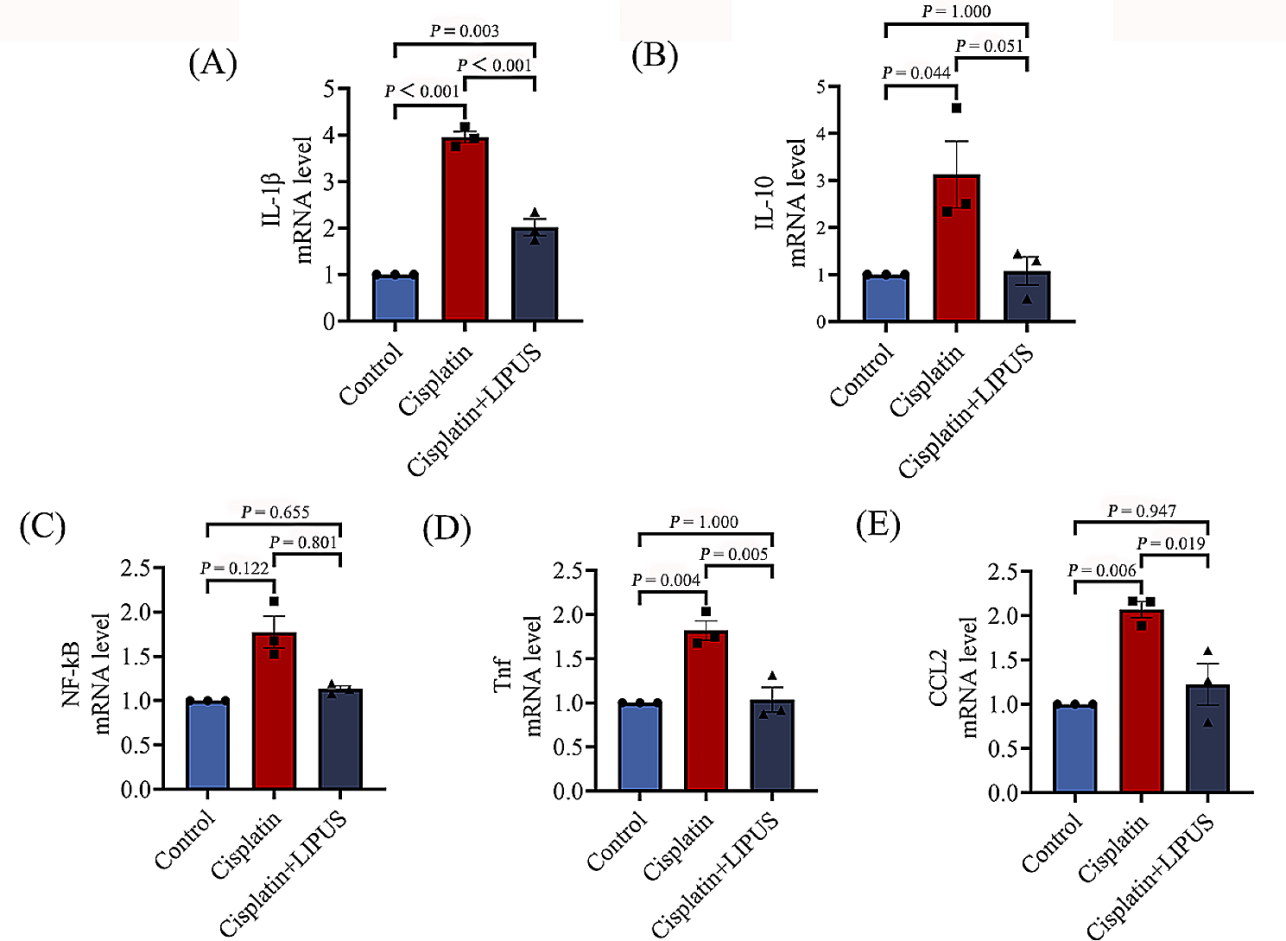
(**A**-**E**): Inflammatory cytokines (*NF-κB, IL-1β, Tnf*, and *Ccl2, IL-10*) mRNA expression levels were assessed by using RT‒qPCR (All results were corrected by Bonferroni method, and *P* < 0.05 after correction was considered to be statistically significant); *n* ≥ 3

Damage caused by oxidative stress can trigger an increased inflammatory response. The ultimate outcome of repeated inflammatory damage is tissue fibrosis [22].To evaluate whether LIPUS can inhibit ovarian inflammation after cisplatin injection in mice, classical inflammatory cytokine mRNA expression levels were assessed by using quantitative real-time reverse transcriptase PCR (RT‒qPCR). The results showed that the levels of *IL-1β, Tnf*, and *Ccl2* in the Cisplatin group were significantly higher than those in the Control group and the Cisplatin + LIPUS group. The Cisplatin + LIPUS group was similar to the Control group (Fig. 8A, D and E). *NF-κB, IL-10* levels did not show significant differences. The results also confirmed that LIPUS can improve ovarian function by reducing inflammation (Fig. 8B-C).

## Discussion

Women in their reproductive years are 38% less likely to become pregnant after cancer chemoradiotherapy [23]. Chemotherapy drugs can cause oocyte and granulosa cell apoptosis while inhibiting tumour cells; moreover, it can eventually lead to irreversible ovarian damage and oestrogen deficiency. In addition to affecting fertility and increasing the risk of early menopause, oestrogen deficiency can have serious adverse effects on other organs, including the bones and cardiovascular and nervous systems [24–26].At present, there is no effective treatment plan in the clinical setting; thus, the protection of the reproductive health of women undergoing chemotherapy has become a problem that must be resolved during the course of chemotherapy. LIPUS has become a significantly researched topic in the field of treatment, we hypothesized that LIPUS can prevent ovarian injury by chemotherapy. Cisplatin is a commonly used as an inducer of premature ovarian failure induced by chemotherapy. We used cisplatin in our research to build animal models. To simulate chemotherapy and the ovarian saving process throughout chemotherapy, mice were intraperitoneally injected with cisplatin every other day, and LIPUS irradiation was performed on the second day of injection for a total of 10 times of LIPUS irradiation. Although some animal experiments and human randomized experiments have proved that validation hormones can well reflect ovarian reserve function, such as: FSH, LH, testosterone, and AMH. Considering the limited venous blood in mice, many indicators and large fluctuations, we directly adopted the follicle count at all levels to verify the success of the establishment of animal models [27–29].The results of ovarian morphology and follicle count after HE staining confirmed the success of our modelling. After cisplatin injection, the ovarian volume was significantly reduced, and the shape was abnormal. Primordial follicles, antral follicles, and secondary follicles were reduced. Moreover, we found that compared with the Cisplatin group, the number of primordial follicles, primary follicles, and secondary follicles in the Cisplatin + LIPUS group increased.

The granulosa cells and membrane cells around the follicle are closely related to the growth, development and release of the follicle. Follicular atresia is caused by granulosa cell atresia of up to 10% [30, 31]. In addition, the proliferation and apoptosis of granulosa cells have become the focus of research on ovarian function. TUNEL assays showed that LIPUS can inhibit the apoptosis of granulosa cells and follicles and maintain the number of follicle pools. To verify our results, apoptosis-related proteins were detected. The most important pathway of apoptosis in granulosa cells is regulated by the bcl-2 family. Bax initiates apoptosis and promotes follicular atresia [32].The results of IHC and WB showed that after chemotherapy injection, the expression of Bax in the ovaries of mice was increased, and it was mainly concentrated in granulosa cells and the follicular fluid. After LIPUS treatment, the expression of Bax was significantly decreased, and granulosa cell apoptosis was decreased. The expression of Bcl-2 by IHC was opposite to that of Bax. This result confirmed that LIPUS can inhibit the apoptosis of mice ovarian granulosa cells caused by chemotherapy drugs.

To understand the transcript information of ovarian tissue in mice treated with different treatments, transcriptome sequencing was conducted, and the differentially expressed genes were analysed. Differential gene cluster analysis showed that the gene expression of the ovarian tissue of mice treated with LIPUS was more similar to that of the Control group, whereas the gene expression of mice treated with chemotherapy alone was significantly different. We performed GO analysis for significantly differentially expressed genes. The differentially expressed genes were mainly concentrated in the following functional regions: integral component of membrane, glutathione transferase activity, growth factor activity, positive regulation of apoptotic process, positive regulation of reactive oxygen species metabolic process, oxidoreductase activity, response to toxic substance, collagen-containing extracellular matrix, cytokine activity, and signal transduction.These also confirmed that LIPUS could reduce ovarian damage caused by chemotherapy drugs and protect ovarian function.

In the biogenic analysis of transcriptome sequencing results, we found differences in fibrosis among the three groups of mice treated with different treatments. It has been reported that granulosa cell degeneration leads to stromal fibroblast hyperplasia, as well as fibrinogen and collagen deposition, thus inducing ovarian fibrosis [33]. Many organ failures are due to fibrosis. Umehara et al. have shown that targeting ovarian fibrosis with pharmaceuticals can extend ovarian ovulatory function [34]. We performed related tests for fibrosis. In the ovarian tissue of mice in the Cisplatin group, positive staining was mainly concentrated in the ovarian stroma, and the positive area and brightness were significantly higher than those in the Control group. Additionally, mice treated with LIPUS exhibited remission. IHC was used to detect the fibrosis markers α-SMA and Collagen I, and the results were consistent with the Masson and Sirus red staining results. The positive intensity of fibrosis marker staining in LIPUS-treated mice was significantly lower than that in untreated mice. Furthermore, we performed quantitative detection of α-SMA protein expression, and the results were consistent with the IHC results. It was verified that LIPUS could inhibit ovarian fibrosis and improve ovarian function in mice treated with chemotherapy.

TGF-β promoted the deposition of extracellular matrix and was the strongest promoter [35]. It can induce the synthesis and deposition of collagen fibers. Its classic pathway is TGF-β1/Smad. By activating smad2 and smad3 and binding to smad4, the expression of target genes is regulated [19]. In order to explore the mechanism of how lLIPUS improves ovarian fibrosis, we detected TGF-β1, p-smad2/3. Immunohistochemical results showed that in terms of brightness intensity and proportion of positive, the Cisplatin group was significantly higher than that of Control and Cisplatin _+_ LiIPUS, positive staining was concentrated around the follicle, in the ovarian stroma. LIPUS was significantly reduced compared with the Cisplatin group, similar to the Control group. We also used Western Blot for protein quantitative analysis, include: TGF-β1, p-smad2, p-smad3, smad2, smad3. The relative level of smad2 was not significantly different between control and treatment groups. The expression of other proteins in the Cisplatin group was significantly higher. After LIPUS treatment, the protein expression decreased significantly, which supports the notion that LIPUS regulates TGF-β1/Smad pathway and inhibits ovarian fibrosis. And now the latest evidence suggests that minimizing ovarian fibrosis could be a strategy to reduce ovarian cancer risk [20]. This suggestion provides a new idea for low intensity ultrasound to inhibit ovarian fibrosis.

It has also been confirmed in the previous literature that the improvement of ovarian fibrosis mainly occurs through the improvement of upstream oxidative damage [20]. Meanwhile, Oxidative stress is an important mechanism of cisplatin toxicity to ovarian cells [4], and it is highly correlated with ovarian function and fecundity. The scavenging of oxygen free radicals can improve reproductive diseases [36, 37]. This process is not only involved in follicular atresia but is also an important cause of granulosa cell apoptosis. We have demonstrated that LIPUS can improve the inhibition of ovarian fibrosis, and our transcriptome biogenic analysis also demonstrated genetic differences in oxidative stress. We verified classical antibodies to oxidative stress by using IHC and WB detection methods. Antibodies against 4HNE, 8OHdG, and 3NIT were detected by IHC. After cisplatin injection, the ovaries of mice undergoing chemotherapy suffered oxidative damage. Moreover, the positive signals were mainly concentrated in granulosa cells and the follicular fluid. We also conducted quantitative detection of protein expression and detected the expression of NOX4. NOX4, which is a major contributor to oxidative stress, is a major source of ROS. The results showed that the protein expression level of mice treated with chemotherapy alone was significantly higher than that of the other two groups. The results of different tests confirmed that LIPUS can alleviate the oxidative damage to ovaries caused by chemotherapy drugs.

Meanwhile, ovarian fibrosis develops in response to repeated tissue insult and inflammation [20]. Pro-inflammatory chemokines recruit immune cells, mainly M1 macrophages, to the injured site, and anti-inflammatory signals induce immune cell differentiation. M2 macrophages then stimulate collagen production via neighboring fibroblasts. Inflammation drives fibrosis [37]. Thus, the inhibition of inflammation can improve ovarian fibrosis [38, 39]. Therefore, we conducted correlation verification of inflammatory factors. The results of RT‒qPCR showed that the expression of inflammatory cytokines in the ovarian tissue damaged by chemotherapy drugs was significantly higher than that in the Control group, and the expression of inflammatory cytokines (*IL-1β, Tnf* and *Ccl2*) decreased after LIPUS treatment. The results also confirmed that LIPUS can improve ovarian function by reducing inflammation.

The main limitation of our study: We verified the specific aspects of LIPUS to improve ovarian damage and the potential mechanism, however, no evidence was provided to substantiate that oocyte quality and fertility were actually improved. We will continue to perform relevant experimental verifications.

## Conclusion

In conclusion, LIPUS can reduce the toxic effect of chemotherapy drugs on ovaries, inhibit ovarian fibrosis through down-regulating TGF-β1/Smad pathway, reduce the inflammatory response, and improve the oxidative damage caused by chemotherapy to reduce follicle depletion and to maintain the number of follicle pools. This method could provide a new idea for tumor patients to reduce the side effects of chemotherapy and to protect ovarian function.

### Electronic supplementary material

Below is the link to the electronic supplementary material.

Supplementary Material 1: Uncropped western blot initial image

## Data Availability

The datasets used and/or analyzed during the current study are available from the corresponding author on reasonable request.
