# Supplementary figures and images for: The value of low-intensity pulsed ultrasound in reducing ovarian injury caused by chemotherapy in mice
Source: Reprod Biol Endocrinol. 2024 Apr 26;22:51. doi: 10.1186/s12958-024-01216-8 (PMC11046824; doi:10.1186/s12958-024-01216-8)

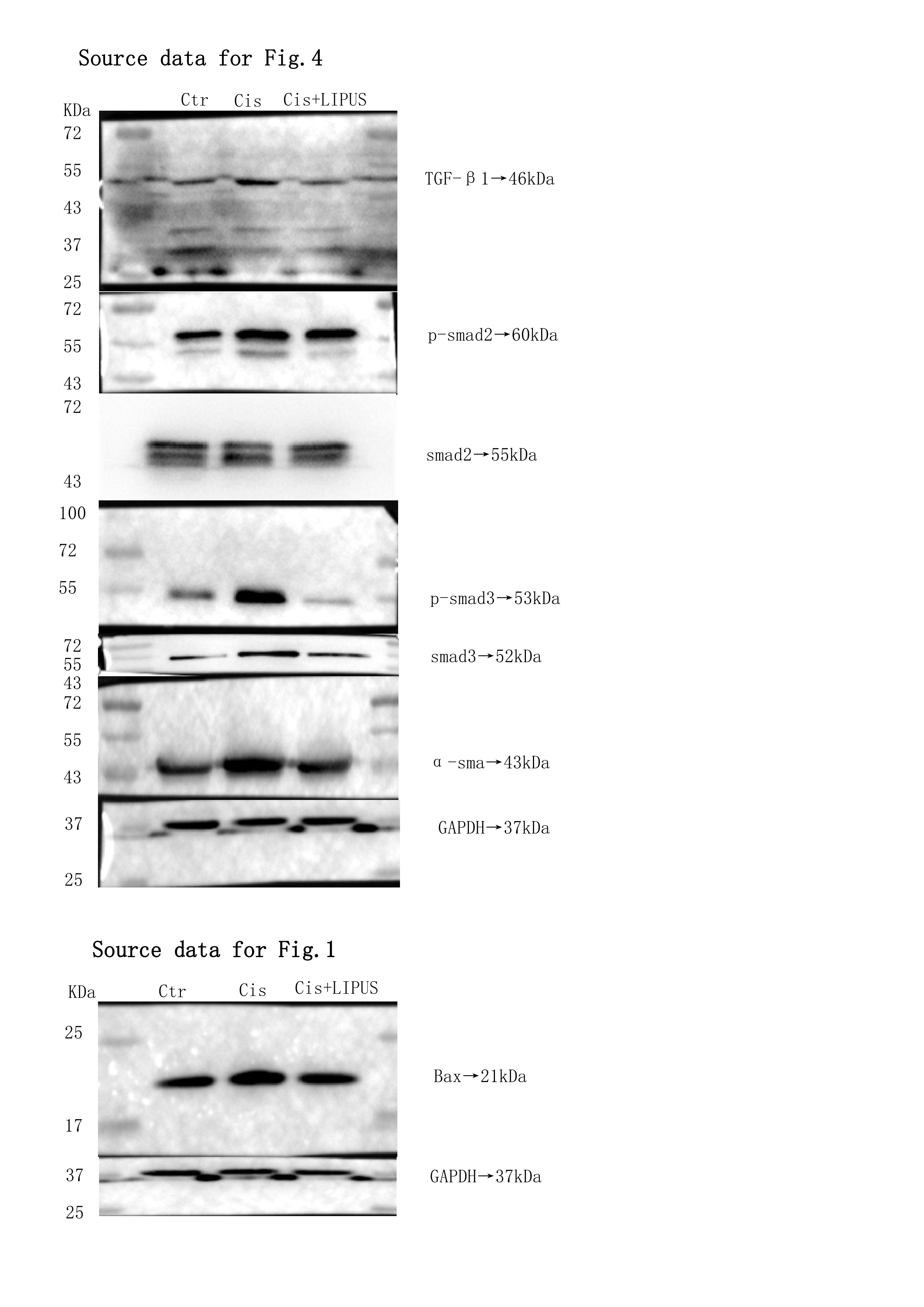

Supplement: Supplementary file 1 — Supplementary Material 1: Uncropped western blot initial image [file 12958_2024_1216_MOESM1_ESM.tif]
